# Grip Strength Changes in Bone Marrow Transplant Patients

**DOI:** 10.1093/geroni/igaa057.1685

**Published:** 2020-12-16

**Authors:** Paulo Chaves, Adrian Cristian, Marco Ruiz

**Affiliations:** 1 Florida International University, Miami, Florida, United States; 2 Baptist Health South Florida, Miami, Florida, United States

## Abstract

Emerging evidence that cancers survivors may experience premature aging has prompted epidemiology research aimed at the characterization of key premature aging phenotypes. Physical functioning data relevant for premature aging phenotype classification in cancer survivors, however, remain scarce. We assessed the burden of dynapenia, a key sarcopenia indicator, and muscle strength loss in the understudied population of bone marrow transplant (BMT) patients. This study involved secondary analyses of data from patients who underwent bone marrow transplant (BMT) in a cancer Institute in South Florida (June 2018-April 2019) with traditional hematopoietic cell transplantation specific comorbidity index (HCT-CI) scores and other relevant information. T-tests for paired data assessed pre- vs. post-BMT changes in grip strength (GS) (n=9). Prevalence of dynapenia (GS<27Kg and <16Kg) in pre-BMT (n=20) and post-BMT (n=9) periods were calculated. Median age [25th-75th percentiles] age was 61 [55-68] years old. Fifty-percent were females. GS and HCT-CI scores were not correlated. Mean GS loss after BMT was 6.4 Kg (95% confidential interval: 2.5-10.4 Kg, p-value=.003) in post-BMT (median-time-after-BMT: 90 days) minus pre-BMT (median-time-before-BMT: 62 days) comparison. Proportion with dynapenia increased from 10% (1/10) to 20% (1/5) in women, and 10% (1/2) to 75% (3/4) in men. We observed substantial muscle strength loss and dynapenia burden in patients who underwent BMT. Considering that dynapenia is a major, potentially modifiable risk factor for frailty, which is not captured by HCT-TI, we speculated that dynapenia-related targeted assessments and interventions – preventive and/or rehabilitative – could offer complementary approaches for treatment enhancement in BMT patients.

